# PODXL might be a new prognostic biomarker in various cancers: a meta-analysis and sequential verification with TCGA datasets

**DOI:** 10.1186/s12885-020-07108-5

**Published:** 2020-07-02

**Authors:** Siying He, Wenjie Du, Menglan Li, Ming Yan, Fang Zheng

**Affiliations:** 1 Center for Gene Diagnosis, Zhongnan Hospital of Wuhan University, Wuhan, Hubei China; 2 Department of Ophthalmology, Aitong Eye Hospital, Maoming, Guangdong China; 3 Department of Ophthalmology, Zhongnan Hospital of Wuhan University, Wuhan, Hubei China

**Keywords:** Cancer, Meta-analysis, Podocalyxin-like protein, Prognosis, TCGA

## Abstract

**Background:**

Several studies have investigated the associations between the podocalyxin-like protein (PODXL) expression quantity or locations and cancers survival, but the results were far from conclusive. Therefore, we proceeded a meta-analysis on PODXL in various human cancers to find its prognostic value and followed confirmation using the TCGA datasets.

**Methods:**

We performed a systematic search, and 18 citations, including 5705 patients were pooled in meta-analysis. The results were verified with TCGA datasets.

**Results:**

Total eligible studies comprised 5705 patients with 10 types of cancer. And the result indicated that PODXL high-expression or membrane-expression were significantly related to poor overall survival (OS). However, subgroup analysis showed a significant association between high expressed PODXL and poor OS in the colorectal cancer, pancreatic cancer, urothelial bladder cancer, renal cell carcinoma and glioblastoma multiforme. Then, we validated the inference using TCGA datasets, and the consistent results were demonstrated in patients with pancreatic cancer, glioblastoma multiforme, gastric cancer, esophageal cancer and lung adenocarcinoma.

**Conclusion:**

The result of meta-analysis showed that high expressed PODXL was significantly linked with poor OS in pancreatic cancer and glioblastoma multiforme, but not in gastric cancer, esophageal cancer or lung adenocarcinoma. And the membrane expression of PODXL might also associate with poor OS. PODXL may act as tumor promotor and may serve as a potential target for antitumor therapy.

## Background

Nowadays, noncommunicable diseases (NCDs) account for the majority of global deaths, and cancer predicts to be the leading cause. According to the latest global cancer statistics, 18.1 million new cancer diagnoses and 9.6 million deaths are expected in 2018 [1].

Podocalyxin-like protein (PODXL) is a highly glycosylated type I transmembrane protein associated with CD34 [2–4]. PODXL expression has been reported in the cytoplasm of some tumor cells, in some cases protruding toward the cell membrane, but not in the nucleus [5]. PODXL is encoded on chromosome 7q32-q33, and highly expressed by glomerular podocytes, vascular endothelium, hematopoietic cells and breast epithelial cells [6–8], which involved in many physiologic processes, such as hematopoiesis [9], leucocyte-endothelial cell interaction [10], regulating vascular permeability [11] and neural development [12].

The clinical significance of PODXL in the progression of various cancers has been studied, and it was found as a stem cell marker in the testicular cancer at the first time [3]. The later findings proved that, PODXL associates with advanced tumor phenotype in some cancers, including breast cancer [1, 13], colorectal cancer [5, 14–16], esophageal cancer [17], gastric cancer [17–19], glioblastoma multiforme [20], lung adenocarcinoma [21], oral squamous cell carcinoma [4, 22], ovarian cancer [23], pancreatic cancer [24–27], prostate cancer [28, 29], renal cell carcinoma [30], urothelial bladder cancer [31], and so on.

In addition, the prognostic role of PODXL protein expression had been analyzed with systematic review and meta-analysis in 2017 [32]. But as new researches emerged, we performed a new meta-analysis at pooling data, in order to estimate the potential prognostic value of PODXL in deep. We explored the relationship between the expression level or site of PODXL and prognosis of multiple cancers. And the validation with the Cancer Genome Atlas (TCGA, http://cancergenome.nih.gov) datasets even had been added for further analysis.

## Methods

### Publication search

Our meta-analysis followed the guidance of the Preferred Reporting Items for Systematic Reviews and Meta-Analysis (PRISMA) [33]. We performed a systematic search of the PubMed, Web of Science, Embase and Cochrane Library database from January 1, 2000 to October 31, 2018, using both MeSH search for keywords and full text. Our search terms were: (“cancer” OR “tumor” OR “neoplasm” OR “carcinoma”) AND (“Podocalyxin like protein” OR “Podocalyxin” OR “PODXL”) AND (“prognosis” OR “prognostic” OR “outcome”). Additionally, the references and other related researches were reviewed to find more potential articles.

### Inclusion and exclusion criteria

The eligible articles selection process was done by two authors (Siying He and Menglan Li). The inclusion criteria were as followed: (1) involved the correlation between the expression of PODXL and survival data of cancer patients; (2) provided the relevant clinicopathological parameters; (3) the number of patients involved in the studies should be more than 50.

The exclusion criteria were as followed: (1) studies that not based on human; (2) insufficient Hazard ratios (HRs) or other data; (3) repetitive patients; (4) reviews, case reports or a meta-analysis.

### Data collection and quality detection

Two researchers evaluated and collected data from these eligible articles with a predefined standard independently. The following information was recorded: (1) first author’s name; (2) publication year; (3) countries; (4) types of cancers; (5) number of patients; (6) detection methods; (7) cut-off criteria; (8) clinical parameters; (9) data about overall survival (OS), disease-free survival (DFS) or cancer-specific survival (CSS). The Engauge Digitizer 4.1 software was used to extract data from Kaplan-Meier (K-M) plot, when there was no HRs and its 95% confidence inter (CIs) offered directly [34]. In addition, the included studies should be evaluated with the Newcastle-Ottawa Scale (NOS) [35].

### Data collection and analysis in TCGA

Data for the expression of PODXL and clinicopathological parameters in TCGA were recorded from the Gene Expression Profiling Interactive Analysis (GEPIA, http://gepia.cancer-pku.cn) [36] and the UALCAN (http://ualcan.path.uab.edu) [37]. There were 31 types of cancer, including 9040 subjects which had both PODXL expression and cancer survival data. In order to make the K-M survival analysis and generated overall survival plots, the expression levels of PODXL were divided into low/median and high expression group according to the TPM value. The difference between two groups was conducted by Log-rank test.

### Mechanism prediction of PODXL

We used the STRING database (http://string-db.org/) [38], online common software, for finding PODXL-related genes and providing a critical assessment and integration of protein-protein interactions (PPI) of PODXL and PODXL-related genes. And these PODXL-related genes were performed functional enrichment analysis by using DAVID database (http://david.abcc.ncifcrf.gov/), which means a common bioinformatics database for annotation, visualization and integrated discovery [39].

### Statistical analysis

Our meta-analysis was based on the Stata12.0 software (Stata Corporation, College Station, TX, United States). The prognostic value of PODXL on OS, DFS and CSS was calculated by pooled HRs with 95% CIs. On the other hand, odds ratios (ORs) with corresponding 95% CIs were used to assess the relation between PODXL and clinicopathological features. Chi square-based Cochran *Q* test and *I*^2^ test were used to determine the heterogeneity among these eligible articles. *I*^2^ > 50% or *P*-value < 0.05 was considered as significant heterogeneity, and a random-effect model would be adopted; otherwise, a fix-effect model would be chose. The effect of covariates have been evaluated with regression analysis. The sources of heterogeneity could be dissect with subgroup analysis. In addition, the sensitivity and publication bias were performed. *P* < 0.05 was considered statistically significant with two-sided.

## Results

### Search results and research characteristics

**Fig. 1:**
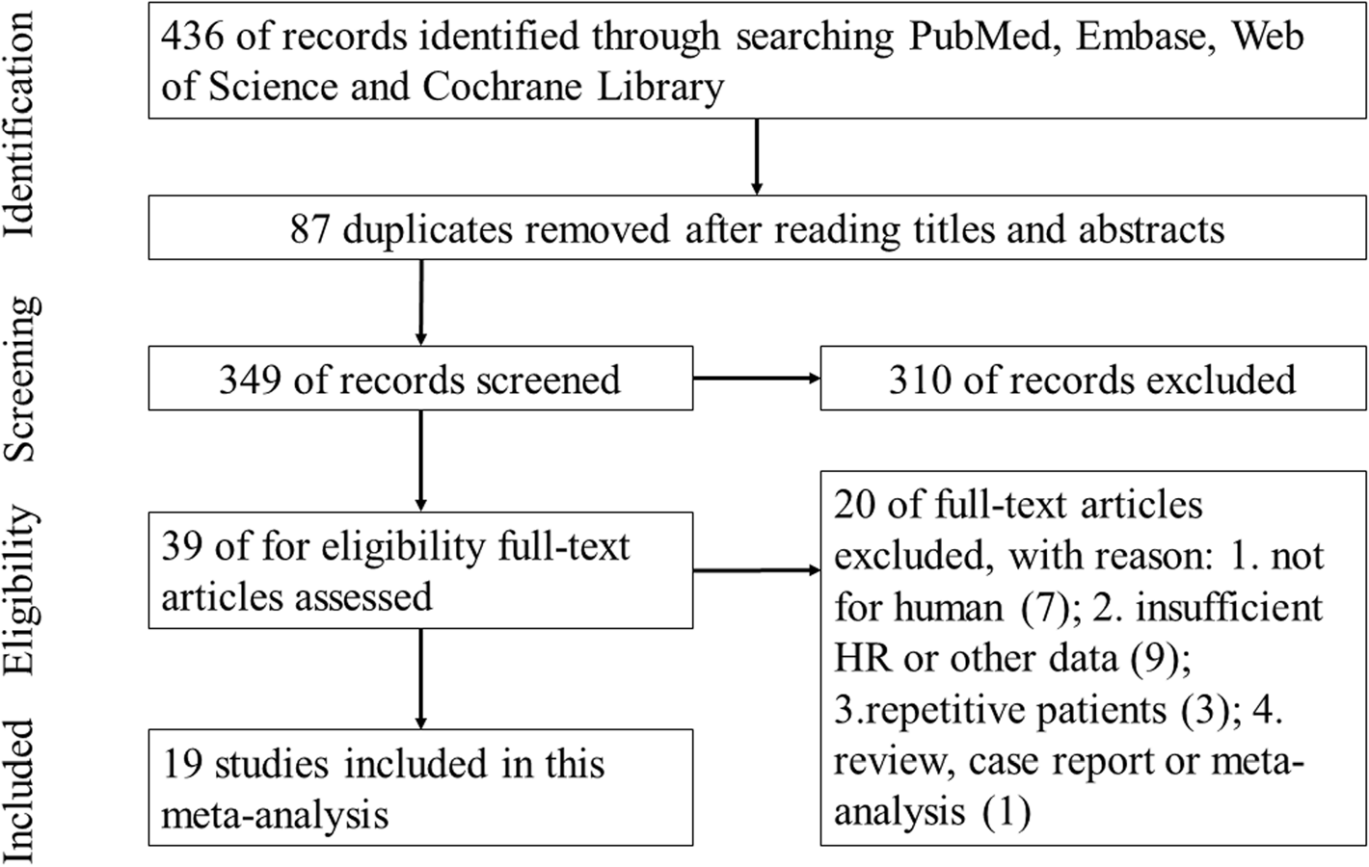
Flow diagram of study selection

In total, 436 records were identified and 87 duplicates were excluded. 39 articles remained after scanning the titles and abstracts, and among the 39 studies, 7 were excluded for not for human, 9 were excluded for insufficient HRs or other data, 3 were excluded because the included patients were repetitive in other studies, and 1 meta-analysis was excluded, and the flow diagram was shown in Fig.1. Finally, 18 eligible studies were include in this meta-analysis [1, 5, 13–21, 23–27, 30, 31].. These eligible researches contained 5705 patients, involved 10 types of cancers, including the breast cancer (*n =* 2), renal cell carcinoma (*n =* 1), colorectal cancer (*n =* 4), ovarian cancer (*n =* 1), glioblastoma multiforme (*n =* 1), urothelial bladder cancer (*n =* 2), pancreatic adenocarcinoma (*n =* 4), esophageal cancer (*n =* 1), gastric cancer (*n =* 3) and lung adenocarcinoma (*n =* 1). In these studies, PODXL expression levels were evaluated by immunohistochemistry (IHC). The characteristics of the eligible articles were listed in Table 1.

**Table 1:** Characteristics of eligible studies in this meta-analysis

Author	Year	Country	No. of Patient	Tumor type	Method	Cut-off	Outcome	Analysis	Antibody	NOS
Somasiri	2004	Canada	272	Breast cancer	IHC	IHC ≥ 50%	CSS	K-M Curve	M	7
Hsu	2010	Taiwan	303	Renal cell carcinoma	IHC	IHC score ≥ 1	OS, CSS, MFS	Multivariate	P	8
Larsson	2011	Sweden	626	Colorectal cancer	IHC	IHC score ≥ 3	OS, CSS	Multivariate	P	8
Cipollone	2012	Canada	479	Ovarian cancer	IHC	IHC score ≥ 1	DFS	K-M Curve	M	8
Larsson	2012	Sweden	607	Colorectal cancer	IHC	IHC score ≥ 3	OS, DFS, TTR	Multivariate	P	9
Binder	2013	America	181	Glioblastoma multiforme	IHC	NA	OS	Multivariate	NA	7
Boman	2013	Sweden	100	Urothelial bladder cancer	IHC	IHC score ≥ 3	OS	Multivariate	M/P	7
Boman	2013	Sweden	343	Urothelial bladder cancer	IHC	IHC score ≥ 3	OS, CSS, PFS	Multivariate	M/P	8
Forse	2013	Canada	698	Breast cancer	IHC	IHC score ≥ 3	DFS	Multivariate	P	9
Kaprio	2014	Finland	840	Colorectal cancer	IHC	IHC score ≥ 3	CSS	K-M Curve	M/P	9
Heby	2015	Sweden	175	Pancreatic and periampullary adenocarcinoma	IHC	IHC score ≥ 2	OS, DFS	Multivariate	P	7
Laitinen	2015	Finland	337	Gastric cancer	IHC	IHC score ≥ 1	CSS	Multivariate	M/P	8
Saukkonen	2015	Finland	189	Pancreatic ductal adenocarcinoma	IHC	IHC score ≥ 3	CSS	Multivariate	M/P	7
Borg	2016	Sweden	106	Esophageal cancer	IHC	IHC score ≥ 1	OS, TTR	K-M Curve	P	7
Borg	2016	Sweden	65	Gastric cancer	IHC	IHC score ≥ 1	OS, TTR	K-M Curve	p	7
Chijiiwa	2016	Japan	70	Pancreatic cancer	IHC	IHC score ≥ 4	OS, DFS	K-M Curve	M	7
Taniuchi	2016	Japan	102	Pancreatic cancer	IHC	IHC score ≥ 3	OS	Multivariate	P	7
Kusumoto	2017	Japan	114	Lung adenocarcinoma	IHC	IHC score ≥ 1	OS, DFS, CSS	K-M Curve	NA	8
Yuan	2018	China	87	Colorectal cancer	IHC	IHC score ≥ 3	OS	Multivariate	M	7
Zhang	2018	China	54	Gastric cancer	IHC	IHC score ≥ 1	OS, DFS	Multivariate	NA	7

*IHC* Immunohistochemistry, *NA* Not Available, *OS* Overall Survival, *DFS* Disease-free Survival, *CSS* Cancer-specific Survival, *NOS* Newcastle-Ottawa Scale

### Meta-analysis of PODXL expression levels and locations on OS/ DFS/ CSS

**Fig. 2:**
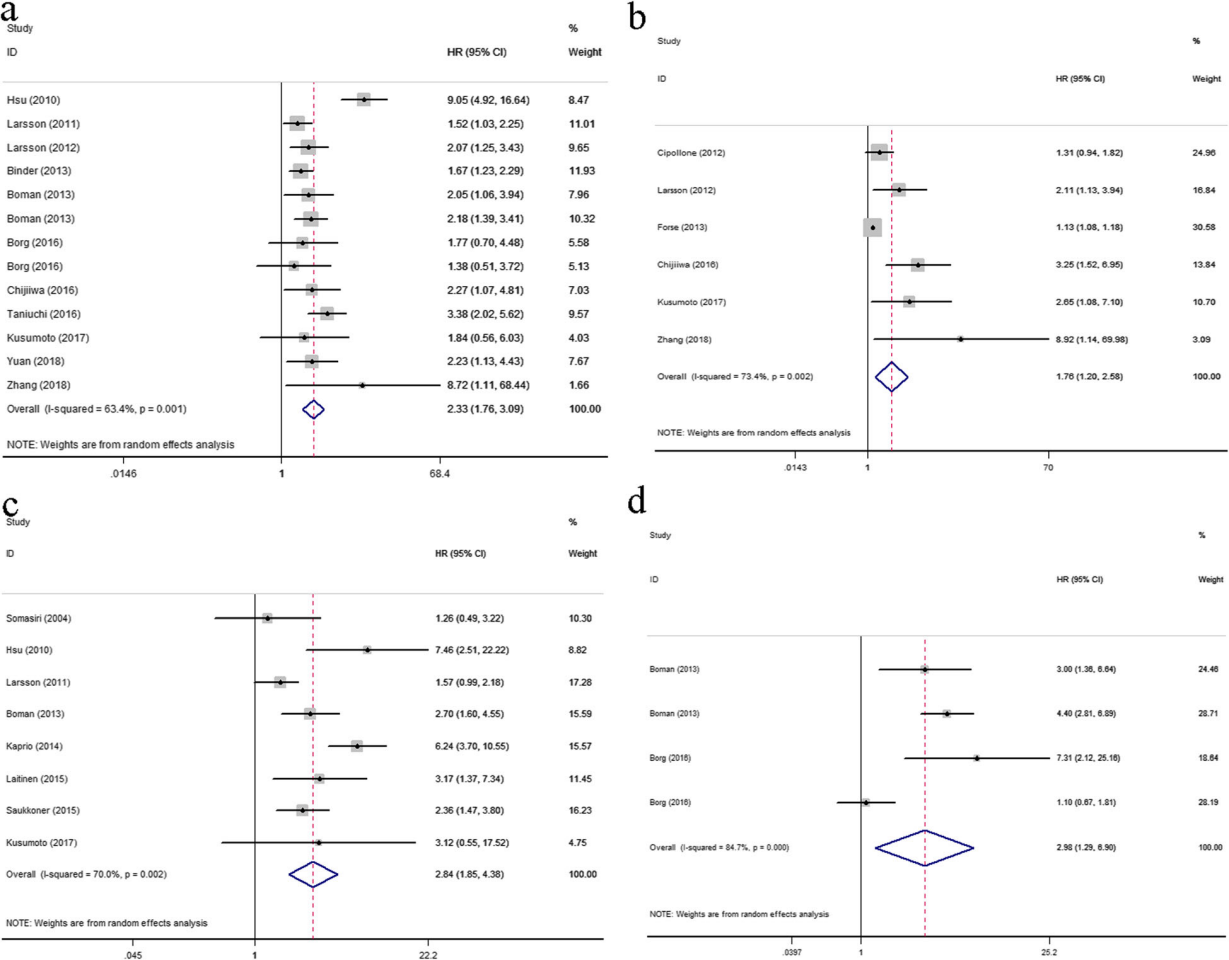
Forest plot of studies evaluating HRs of PODXL expression and the prognosis of cancer patients. **a** High expressed PODXL and the OS; **b** high expressed PODXL and the DFS; **c** high expressed PODXL and the CSS; **d** membrane expressed PODXL and the OS

A total of 11 eligible studies, including 13 cohorts and 2272 patients, were recruited to evaluate the expression level of PODXL on OS. The pooled HR and 95% CI indicated that high-expressed PODXL was significantly related to poor OS in patients with various cancers (HR = 2.33, 95% CI = 1.76–3.09, *P* < 0.0001) with a significant heterogeneity across these studies (*I*^2^ = 63.4%, *P* = 0.001) (Fig.2a). In addition, there were 6 studies performed the relationships between PODXL expression levels and DFS, and 8 studies investigated the associations between PODXL expression levels and CSS respectively. Heterogeneity test indicated both the DFS (*I*^2^ = 73.4%, *P* = 0.002) and CSS (*I*^2^ = 70.0%, *P* = 0.002) should be analyzed using the random-effect model. Finally, the results indicated the association between the high expressed PODXL and the shorter DFS (HR = 1.76, 95% CI =1.20–2.58, *P* = 0.004) or the shorter CSS (HR = 2.84, 95% CI = 1.85–4.38, *P* < 0.0001) (Fig.2b-c). On the other hand, among these eligible 18 papers, 5 studies involved the expression locations of PODXL and the prognosis of cancers, and only 2 studies, including 4 cohorts, showed the association between membrane expressed PODXL and poor OS (HR = 2.98, 95% CI =1.29–6.90, *P* = 0.011), also by using the random-effect model (*I*^2^ = 84.7%, *P* < 0.0001) (Fig.2d).

### Subgroup analysis for OS

In order to find the source of heterogeneity, the subgroup analysis of OS was performed, and all of the 2272 patients were classified based on cancer types, analysis types, antibody types, ethnicities and sample sizes (Table 2). Single study which assessed the relationship between the expression and OS in renal cell carcinoma, glioblastoma multiforme, esophageal cancers and lung adenocarcinoma were defined as “other cancers” in the other cancers subgroup. Subgroup analysis showed that, high expressed PODXL were linked with poor OS in colorectal cancer (HR = 1.79, 95% CI = 1.35–2.37, *P* < 0.0001), pancreatic cancer (HR = 2.98, 95% CI = 1.95–4.55, *P* < 0.0001), urothelial bladder cancer (HR = 2.14, 95% CI = 1.48–3.10) and other cancers (HR = 2.60, 95% CI = 1.45–4.66, *P* = 0.001), but not in patients with the gastric cancer (HR = 2.76, 95% CI = 0.45–15.84, *P* = 0.256). In conclusion, high expressed level of PODXL was associated with poor OS in 6 types of cancers.

**Table 2:** Subgroup analysis of pooled HR for OS

Categories	No. of studies	No. of patients	Pooled HR (95%CI)		Heterogeneity	
			Fix/Random	*P*-value	*I*^*2*^ (%)	*P*-value
OS	13	2272	2.33 (1.76, 3.09)	0	63.4	0.001
Cancer type	8					
Colorectal cancer	3	834	1.79 (1.35, 2.37)	0	0	0.499
Pancreatic cancer	2	172	2.98 (1.95, 4.55)	0	0	0.391
Gastric cancer	2	119	2.76 (0.48, 15.84)	0.256	59.9	0.114
Urothelial bladder cancer	2	443	2.14 (1.48, 3.10)	0	0	0.880
Other cancers	4	704	2.60 (1.45, 4.66)	0.001	83.3	0
Analysis						
K-M curve	4	355	1.85 (1.17, 2.95)	0.009	0	0.89
Multivariate	9	2017	2.59 (1.77, 3.80)	0	74.7	0
Antibody type						
Monoclonal antibody	2	157	2.25 (1.36, 3.73)	0.002	0	0.975
Polyclonal antibody	6	1672	2.55 (1.45, 4.50)	0.001	0	81.6
M + P	2	443	2.14 (1.48, 3.10)	0	0	0.880
Ethnicity						
European	6	1361	1.84 (1.47, 2.30)	0	0	0.834
Asian	6	730	3.49 (2.02, 6.02)	0	64.5	0.015
North American	1	181	1.67 (1.23, 2.29)		–	–
Sample size						
< 150	8	628	2.46 (1.81, 3.33)	0	0	0.536
≥150	5	1644	2.36 (1.53, 3.65)	0	81.3	0

*OS* overall survival, *HR* hazard ratio

And regarding the analysis type, we also found that the high expression of PODXL was significantly associated with the much shorter OS, when the studies were assessed with K-M curve. In the subgroups based on ethnicities, antibody types and sample sizes, we also found that, the relation between high expression level of PODXL and poor OS, except for patients from Asia or the sample size ≥150.

### PODXL overexpression and relative clinical parameters

In order to obtain more clinical values of PODXL, we investigated the associations between PODXL expression levels and clinical parameters in several cancers (Table 3). From these results, we found that the expression level of PODXL was related with the TNM stage (HR = 1.63, 95% CI = 1.19–2.23, *P* = 0.002, fixed-effects), tumor grade (HR = 4.29, 95% CI = 1.84–9.99, *P* = 0.001, random-effects), differentiation (HR = 2.84, 95% CI = 1.82–4.42, *P* < 0.0001, fixed-effects), distant metastasis (HR = 5.46, 95% CI = 2.55–11.66, *P* < 0.0001, fixed-effects), lymph node metastasis (HR = 1.51, 95% CI = 1.03–2.22, *P* = 0.034, fixed-effects), neural invasion (HR = 2.43, 95% CI = 1.02–5.79, *P* = 0 .45, fixed-effects) and vascular invasion (HR = 2.27, 95% CI = 1.56–3.30, *P* < 0.0001, fixed-effects) significantly. Whereas, there was no significant correlations between PODXL expression and age (HR = 0.88, 95% CI = 0.71–1.10, *P* = 0.269, fixed-effects), gender (HR = 1.04, 95% CI = 0.82–1.32, *P* = 0.749, fix-effects) and tumor size (HR = 0.90, 95% CI = 0.61–1.34, *P* = 0.614, fixed-effects). As a result, these correlations indicated that the high expressed PODXL was associated with the advanced biological behavior in various cancers. No covariate analyzed in this study had a statistically significant effect on degree of tumor malignancy and survival.

**Table 3:** Clinicopathological features of the enrolled studies with high expressed PODXL in patients with cancer

Clinicopathological parameters	Studies	No. of patients	Risk of high PODXL OR (95% CI)	Significant Z	*P*-value	Heterogeneity *I*^*2*^ (%)	*P*-value	Model
Age (< 65 vs ≥ 65)	10	2905	0.88 (0.71, 1.10)	1.11	0.269	42.6	0.084	Fixed effects
Gender (male vs female)	11	3081	1.04 (0.82, 1.32)	0.32	0.749	0	0.835	Fixed effects
Tumor size (< 5 cm vs ≥5 cm)	5	1334	0.90 (0.61, 1.34)	0.50	0.614	0	0.703	Fixed effects
TNM stage (III-IV vs I-II)	12	2417	1.63 (1.19, 2.23)	3.04	0.002	13.1	0.319	Fixed effects
Tumor grade (3–4 vs 1–2)	6	2268	4.29 (1.84, 9.99)	3.38	0.001	78.6	0	Random effects
Tumor differentiation (moderate/well vs poor)	6	1429	2.84 (1.82, 4.42)	4.62	0	0	0.559	Fixed effects
Distant metastasis (positive vs Negative)	3	475	5.46 (2.55, 11.66)	4.38	0	44.5	0.165	Fixed effects
Lymph node metastasis (positive vs negative)	6	1574	1.51 (1.03, 2.22)	2.11	0.034	0	0.614	Fixed effects
Neural invasion (positive vs negative)	3	264	2.43 (1.02, 5.79)	2.00	0.045	0	1.000	Fixed effects
Vascular invasion (positive or negative)	6	1240	2.27 (1.56, 3.30)	4.29	0	2.1	0.403	Fixed effects

### Sensitivity analysis and publication bias

**Fig. 3:**
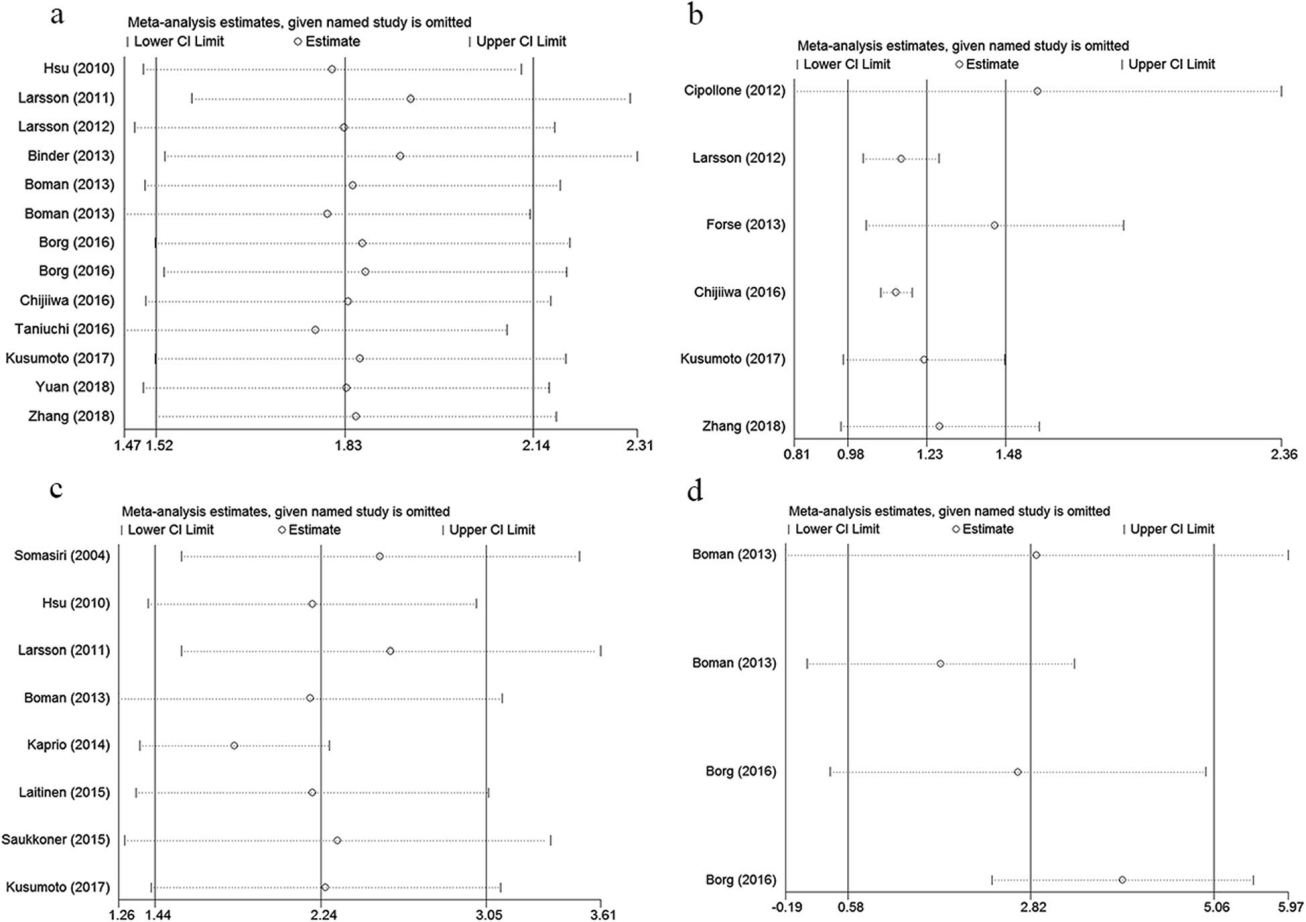
Sensitivity analysis of this meta-analysis. **a** OS of PODXL expression levels; **b** DFS of PODXL expression levels; **c** CSS of PODXL expression levels; **d** OS of PODXL expression locations

**Fig. 4:**
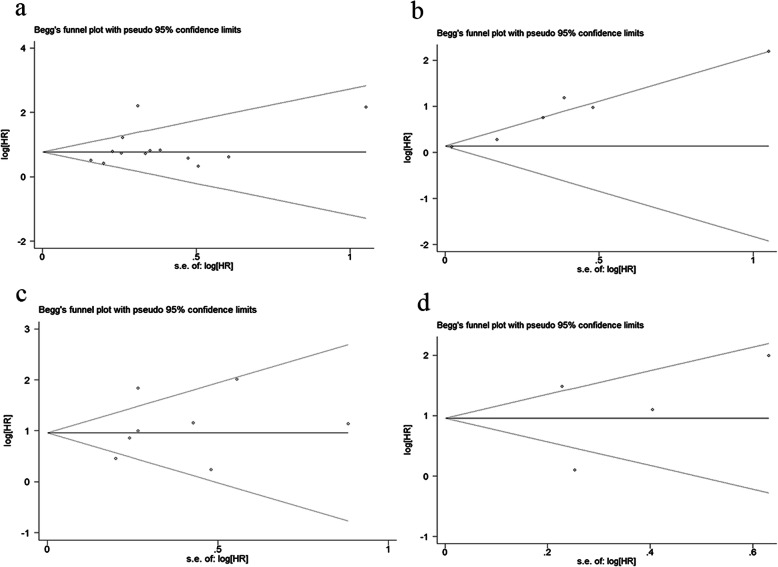
Begg’s funnel plots for the studies involved in the meta-analysis. **a** OS of PODXL expression levels; **b** DFS of PODXL expression levels; **c** CSS of PODXL expression levels; **d** OS of PODXL expression locations

We performed sensitivity analysis to determine whether an individual study could affected the overall result. Results of association studies between PODXL expression and OS and CSS demonstrated that single study had no influence on the result of meta-analysis (Fig.3). Funnel plots and Begg’s test were performed and the results showed no publication bias existed in studies on associations between PODXL overexpression and OS (*P* = 0.502), DFS (*P* = 0.133) and CSS (*P* = 0.266). And no publication bias existed in our meta-analysis on associations between PODXL membrane expression and OS (*P* = 1.000) as well (Fig.4).

### The expression data of PODXL extracted from TCGA datasets

The differences of PODXL expression level between various tumor tissues and corresponding normal tissues were obtained with GEPIA, which was a common web-based tool that can provide a quick and customizable survey of function based on TCGA and GTEx data [36]. PODXL was detected in 23 types of cancers, and the result that the PODXL expression was significantly much higher than the corresponding normal tissues was found in 9 types of cancers, including the esophagus cancer, glioblastoma multiforme, acute myeloid leukemia, liver hepatocellular carcinoma, ovarian serous cystadenocarcinoma, pancreatic adenocarcinoma, rectum adenocarcinoma, stomach adenocarcinoma, testicular germ cell tumor (Table 4).

**Table 4:** The difference of PODXL expression in cancers and corresponding normal tissues in TCGA datasets

Types of cancer	TCGA dataset	No. of cancer tissues	No. of normal tissues	Log2(FC)	*P* value
Adenoid cystic carcinoma	ACC	77	128	− 1.068	1.10e-10
Breast invasion carcinoma	BRCA	1085	291	−0.514	4.01e-16
Cervical squamous cell carcinoma	CESC	306	13	−0.590	0.191
Esophagus cancer	ESCA	182	286	1.391	5.97e-22
Glioblastoma multiforme	GBM	163	207	0.866	1.12e-10
Head and neck squamous cell carcinoma	HNSCC	519	44	0.656	0.123
Kidney chromophobe	KICH	66	53	−2.863	1.53e-10
Kidney renal clear cell carcinoma	KIRC	523	100	−0.732	1.85e-9
Kidney renal papillary cell carcinoma	KIRP	286	60	−4.247	1.31e-43
Acute myeloid leukemia	LAML	173	70	1.210	3.26e-2
Liver hepatocellular carcinoma	LIHC	369	160	1.508	8.20e-32
Lung adenocarcinoma	LUAD	483	347	−2.064	6.27e-122
Lung squamous cell carcinoma	LUSC	486	338	−2.832	3.86e-153
Ovarian serous cystadenocarcinoma	OVSC	426	88	1.449	3.96e-14
Pancreatic adenocarcinoma	PAAD	179	171	0.492	4.05e-5
Prostate carcinoma	PRAD	492	152	−0.479	0.044
Rectum adenocarcinoma	READ	92	318	0.598	8.17e-8
Skin cutaneous melanoma	SKCM	461	558	−0.636	3.83e-6
Stomach adenocarcinoma	STAD	408	211	1.597	1.64e-49
Testicular germ cell tumor	TGCT	137	165	2.750	3.93e-30
Thyroid carcinoma	THCA	512	337	−0.796	6.95e-22
Uterine corpus endometrial carcinoma	UCEC	174	91	−0.797	2.97e-5
Uterine carcinosarcoma	UCS	58	78	−2.075	3.79e-12

### Validation of prognostic correlation by TCGA datasets

**Fig. 5:**
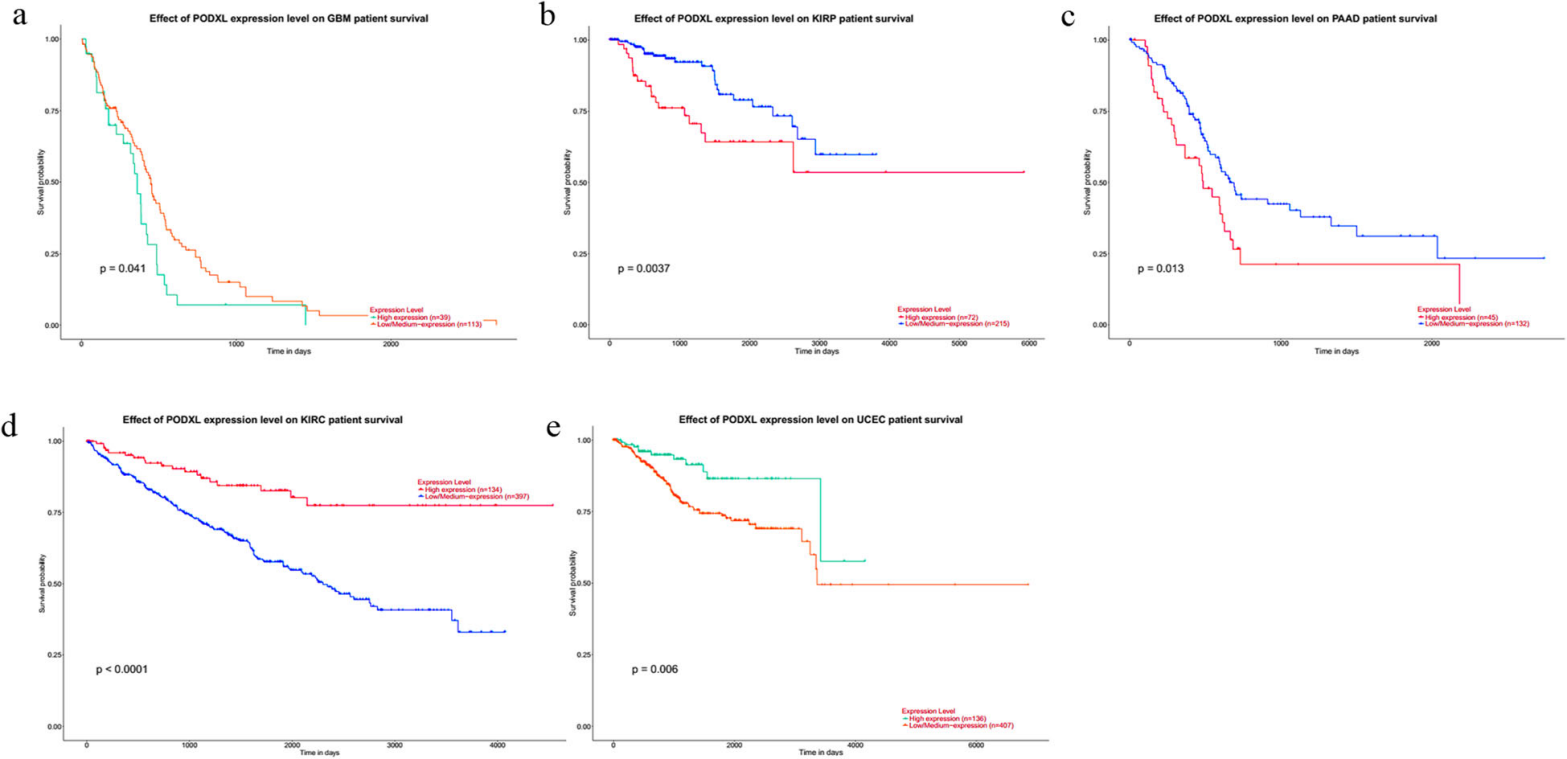
Kaplan-Meier survival curves for cancer patients based on TCGA datasets. **a** glioblastoma multiforme; **b** kidney renal papillary cell carcinoma; **c** pancreatic adenocarcinoma; **d** kidney renal clear cell carcinoma; **e** uterine corpus endometrial carcinoma

To validate the clinical prognosis indication value of PODXL, we explored TCGA datasets by using UALCAN, which was an interactive online tool that could analyze the expression data of genes in TCGA [37]. And among the 31 types of cancers, 9040 patients, the significant association between high expressed PODXL and poor OS was found in 3 types of cancers, including the glioblastoma multiforme, kidney renal papillary cell carcinoma and pancreatic adenocarcinoma (Table 5). But there were adverse results in kidney renal clear cell carcinoma and uterine corpus endometrial carcinoma, which showed a significant correlation between the low expressed PODXL and poor OS (Fig.5). The same results were also verified with KM Plotter, whose data sources were not completely consistent with TCGA datasets (Supplementary Fig. 1, SF.1).

**Table 5:** The difference of overall survival in cancer patients with high PODXL expression vs low/median expression

Cancer type	No. of cancer tissues			*P* value
	High	Low/Median	Total	
ACC	20	59	79	0.37
BLCA	102	304	406	0.34
BRCA	272	809	1081	0.4
CESC	73	218	291	0.77
CHOL	9	27	36	0.57
COAD	69	210	279	0.32
ESCA	46	138	184	0.16
GBM	39	113	152	0.041
HNSCC	130	389	519	0.3
KICH	15	49	64	0.35
KIRC	134	397	531	< 0.0001
KIRP	72	215	287	0.0037
LAML	43	120	163	0.64
LIHC	93	272	365	0.82
LUAD	125	377	502	0.37
LUSC	126	368	494	0.33
DLBC	12	35	47	0.21
MESO	22	63	85	0.23
OVSC	76	227	303	0.95
PAAD	45	132	177	0.013
PCPG	45	134	179	0.13
PRAD	125	372	497	0.92
READ	42	123	165	0.44
SARC	65	194	259	0.12
SKCM	115	344	459	0.22
TGCT	34	100	134	0.29
THYM	30	89	119	0.78
THCA	127	377	504	0.87
UCS	15	41	56	0.58
UCEC	136	407	543	0.006
UVM	20	60	80	0.36

*ACC* adrenocortical carcinoma, *BLCA* bladder urothelial carcinoma, *BRCA* breast invasion carcinoma, *CESE* cervical squamous cell carcinoma, *CHOL* cholangiocarcinoma, *COAD* colon adenocarcinoma, *ESCA* esophageal carcinoma, *GBM* glioblastoma multiforme, *HNSCC* head and neck squamous cell carcinoma, *KICH* kidney chromophobe, *KIRC* kidney renal clear cell carcinoma, *KIRP* kidney renal papillary cell carcinoma, *LAML* acute myeloid leukemia, *LIHC* liver hepatocellular carcinoma, *LUAD* lung adenocarcinoma, *LUSC* lung squamous cell carcinoma, *DLBC* lymphoid neoplasm diffuse large B-cell lymphoma, *MESO* mesothelioma, *OVSC* ovarian serous cystadenocarcinoma, *PAAD* pancreatic adenocarcinoma, *PCPG* pheochromocytoma and paraganglioma, *PRAD* prostate adenocarcinoma, *READ* rectum adenocarcinoma, *SARC* sarcoma, *SKCM* skin cutaneous melanoma, *STAD* stomach adenocarcinoma, *TGCT* testicular germ cell tumors, *THYM* thymoma, *THCA* thyroid carcinoma, *UCS* uterine carcinosarcoma, *UCEC* uterine corpus endometrial carcinoma, *UVM* uveal melanoma

A joint result of our meta-analysis and TCGA datasets validation identified the correlation between the expression level of PODXL and the glioblastoma multiforme, pancreatic adenocarcinoma, esophagus cancer, gastric cancer and lung adenocarcinoma.

### PPI network construction and functional enrichment analysis

**Fig. 6:**
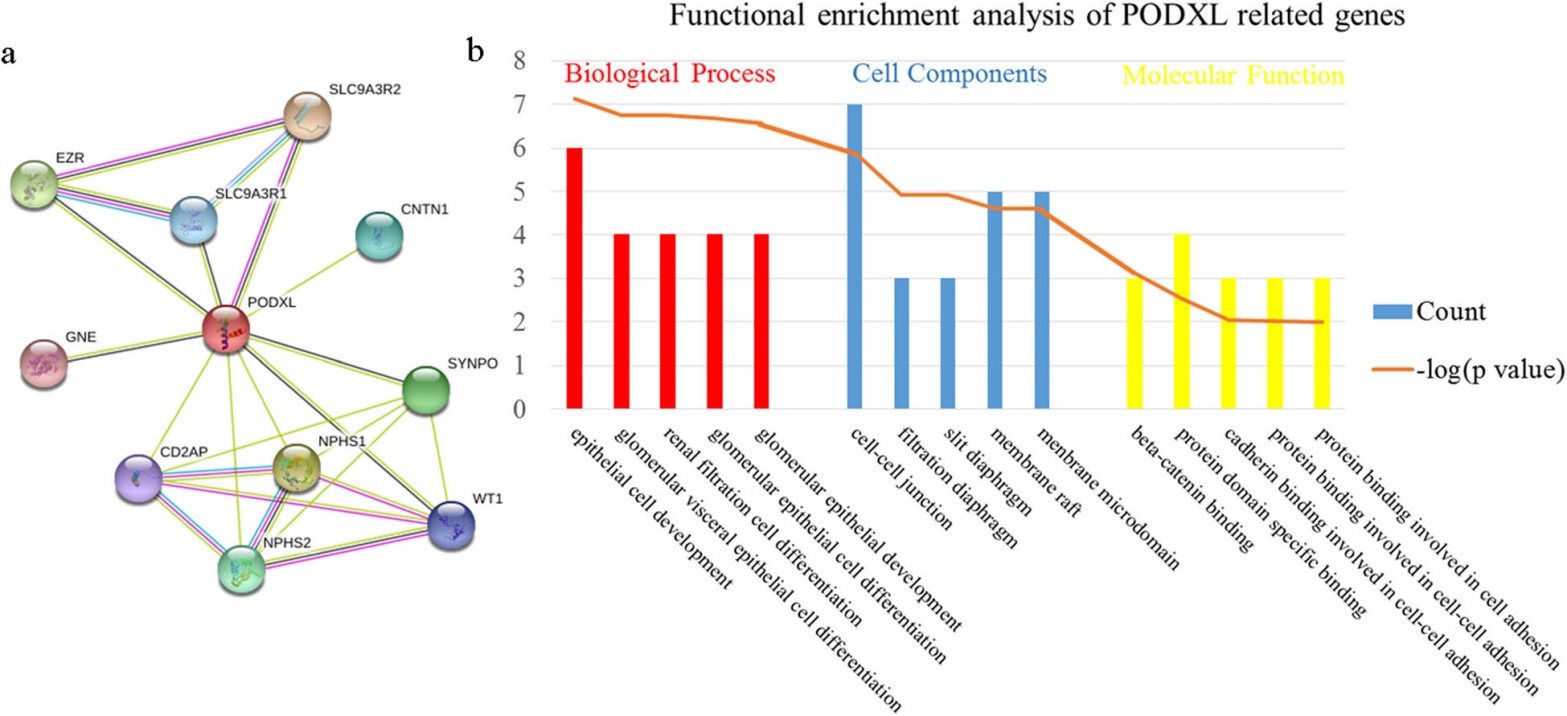
Mechanism prediction of PODXL-related genes with bioinformatics. **a** The protein-protein interaction network of PODXL-related genes. The lines represented the interaction between the nodes. **b** The functional enrichment analysis of PODXL-related genes

The PPI network of PODXL-related genes was obtained by using STRING, including 11 nodes and 23 edges (Fig.6a). The PODXL-related genes were collected for functional enrichment analysis (Fig.6b). The top GO terms, containing biological processes, cell components and molecular function, were selected based on the most significant. These PODXL-related genes were significantly enriched in cell development and differentiation, and played a significant role in cell-cell adhesion. These significant GO terms were matched with the pathogenesis of cancers, such as intercellular adhesion decrease, epithelial-mesenchymal transition (EMT), cell migration and invasion.

## Discussion

Recently, increasing evidences have suggested that PODXL was involved in multiple links in several process of tumor development, such as cell adhesion and morphology [40], lymphatic metastasis [41], tumor cells motility and invasiveness [26], tumor angiogenesis [42] and prognosis. Recent researches indicated that the expression level and location of PODXL could be a new biomarker to assess the prognosis of various types of cancers. However, a single study is limited by insufficient data and single experimental model, so that a meta-analysis of pooling studies is necessary to explore the potential clinical value of PODXL.

Among these published studies, there were 10 types of cancers, including 5705 patients. Our meta-analysis not only indicated that high expressed PODXL was associated with poor OS, DFS or CSS in patients with cancers, but also showed that membrane expression was correlated with poor OS as well. Clinicopathological features analysis showed that the overexpressed PODXL was linked with poor stage and differentiation, and high incidences of metastasis and invasion in cancers, which indicated that there might be a significant association between PODXL expression level and advanced features of cancer. Subgroup analysis showed that the association between overexpressed PODXL and poor OS in patients with cancers, was only significative in the glioblastoma multiforme, pancreatic cancer, renal cell carcinoma, colorectal cancer and urothelial bladder cancer, but not in the esophageal cancer, gastric cancer and lung adenocarcinoma. Then we used GEPIA and UALCAN to explore TCGA datasets, to compare the expression difference of PODXL among tumor tissues and correlated normal tissues, and the survival curves. Consistent results of meta-analysis and TCGA datasets validation were found in 5 types of cancers. Beside TGGA datasets, Oncomine was used to further verify the differences of PODXL expression level between various tumor tissues and corresponding normal tissues. And On the other hand, KM Plotter was used to validate the clinical prognosis indication value of PODXL. The results of these databases also supported the consequence of TCGA datasets.

The prognostic value of PODXL had been indicated by meta-analysis in 2017 [32], the conclusion put forward by Wang et al. was approximately consistent with our results. But we revisited and gathered relevant research for another meta-analysis, in order to further explore its clinical significance. Compared with the meta-analysis in 2017, our research contained more studies and patients, which reinforced the conclusion. In addition, both of the expression level and site of PODXL were found to be associated with prognosis of various cancers. And the results of meta-analysis were filtrated by validation with TCGA datasets, which made our conclusion seem more convincing.

Among the eligible 18 studies, there were only 2 researches mentioned the expression location of PODXL and prognosis of cancers, containing 4 cohorts. The studies showed a significant association between membrane expression of PODXL and poor OS, but the sensitivity analysis showed that this result is not credible. On the premise of appropriate number of included studies, samples that may introduce heterogeneity are moved, but the sensitivity is still high, so this result can only be used as a descriptive hypothesis, and need more included studies. As PODXL is a transmembrane glycoprotein, whose high expression level and membrane expression lead to cell motility increasing, and over-activated tumor cell migration ability promotes tumor progression. Combined with the existing results, the expression site of PODXL was a promising markers in predicting the prognosis of cancers.

Although, PODXL has been found to be highly expressed in various malignancies and was related to a more aggressive phenotype and poor prognosis, the exact mechanisms of which role did PODXL play in tumorigenesis remains unclear [43]. The gene functional enrichment analysis showed that PODXL was a fatal gene in cell development and differentiation, which played an important role in cell-cell adhesion. Some latest studies showed that PODXL promoted the gelsolin-actin interaction in cell protrusions to enhance the motility and invasiveness [26], and some showed that the PODXL-ezrin signaling axis could rearrange the dynamic cytoskeleton for transendothelial migration [44]. According to these reports, it could be deduced that high expressed PODXL promoted tumor progression by enhancing a series of cell changes such as EMT, cell migration and invasion. In addition, the result that membrane-expressed PODXL was associated with poor survival, further supported the deduction that PODXL promoted tumor progression by enhancing the motility and invasiveness of tumor cells. PODXL also took part in the NF-kB, PI3K/AKT, Hippo and MAPK/ERK signaling pathway, and facilitated tumor progression by increasing cell proliferation, migration and invasion as well as suppressing apoptosis [21, 45, 46].

PODXL was expected to be a novel therapeutic and monitoring biomarker in certain cancers, because the high expressed PODXL might be a potential indicator of poor prognosis of cancers. Overexpressed PODXL could be detected in peripheral blood and used as a non-invasive diagnostic biomarker for the detection of pancreatic cancer [47]. ATF3 could activate PODXL transcription, which suggested that ATF3 pathway might be beneficial for anticancer therapy [48]. High expression of miR-509-3-5p and miR-5100 inhibited the invasion and metastasis of gastric cancers and pancreatic cancers by directly targeting PODXL, functioning as a tumor suppressor [27, 41]. A core fucose-deficient monoclonal antibody (mAb) of PODXL might be a new antibody-based therapy method against PODXL high-expressed oral squamous cell carcinoma [49]. And patients with gastric or esophageal adenocarcinoma would have a much better prognosis after treating with neoadjuvant ± adjuvant fluoropyrimidine– and oxaliplantin-based chemotherapy, if the expression level of PODXL is high [50].

However, there are still some limitations. First of all, many unavoidable reasons, such as different types of cancers, the analysis methods, ethnicities and sample sizes might attribute to the heterogeneity. Secondly, we extracted the data of HRs and 95% CIs from the K-M plots when it could not be obtained from the paper directly, and this process might decrease the accuracy of results. Thirdly, the sensitivity analysis only showed that individual study had no influence on the association study between the high expressed PODXL and poor OS or CSS, that is to say, the results of the association between the membrane expressed PODXL and poor OS in cancers can only be seen as a descriptive hypothesis, might be induced by the insufficient studies or the small sample size. Fourthly, our meta-analysis seemed have no publication bias, but as the chance of negative results being published is very small, more studies are needed to verify the results of our meta-analysis.

## Conclusion

PODXL is a significant clinical indicator for tumor prognosis and detection, the expression level and location in tumor tissues, and even the serum concentration of which could be associated significantly with tumor progression [47]. Our meta-analysis showed that PODXL plays a significant role in cancer progression, and high-expressed PODXL could be linked to aggressive biological phenotype and poor prognosis. Specifically, the high expressed PODXL was correlated with poor prognosis significantly in the glioblastoma multiforme and pancreatic cancer, but not in the esophageal adenocarcinoma, gastric cancer and lung adenocarcinoma.

## Supplementary information

**Additional file 1.**

**Additional file 2 SF.1** Kaplan-Meier survival curves for cancer patients from KM Plotter. (a) Pancreatic adenocarcinoma; (b) kidney renal papillary cell carcinoma; (c) kidney renal clear cell carcinoma; (d) uterine corpus endometrial carcinoma.

## Data Availability

All data generated or analyzed during this study are included in this article and referenced articles are listed in the References section.

## Abbreviations

CI: Confidence inter
CSS: Cancer-specific survival
DFS: Disease-free survival
GEPIA: Gene Expression Profiling Interactive Analysis
HR: Hazard ratio
IHC: Immunohistochemistry
K-M: Kaplan-Meier
mAb: Monoclonal antibody
NCD: Noncommunicable disease
NOS: Newcastle-Ottawa Scale
OR: Odds ratio
OS: Overall survival
PRISMA: Preferred Reporting Items for Systematic Reviews and Meta-Analysis
PODXL: Podocalyxin-like protein
TCGA: The Cancer Genome Atlas
