# Supplementary figures and images for: PODXL might be a new prognostic biomarker in various cancers: a meta-analysis and sequential verification with TCGA datasets
Source: BMC Cancer. 2020 Jul 2;20:620. doi: 10.1186/s12885-020-07108-5 (PMC7331259; doi:10.1186/s12885-020-07108-5)

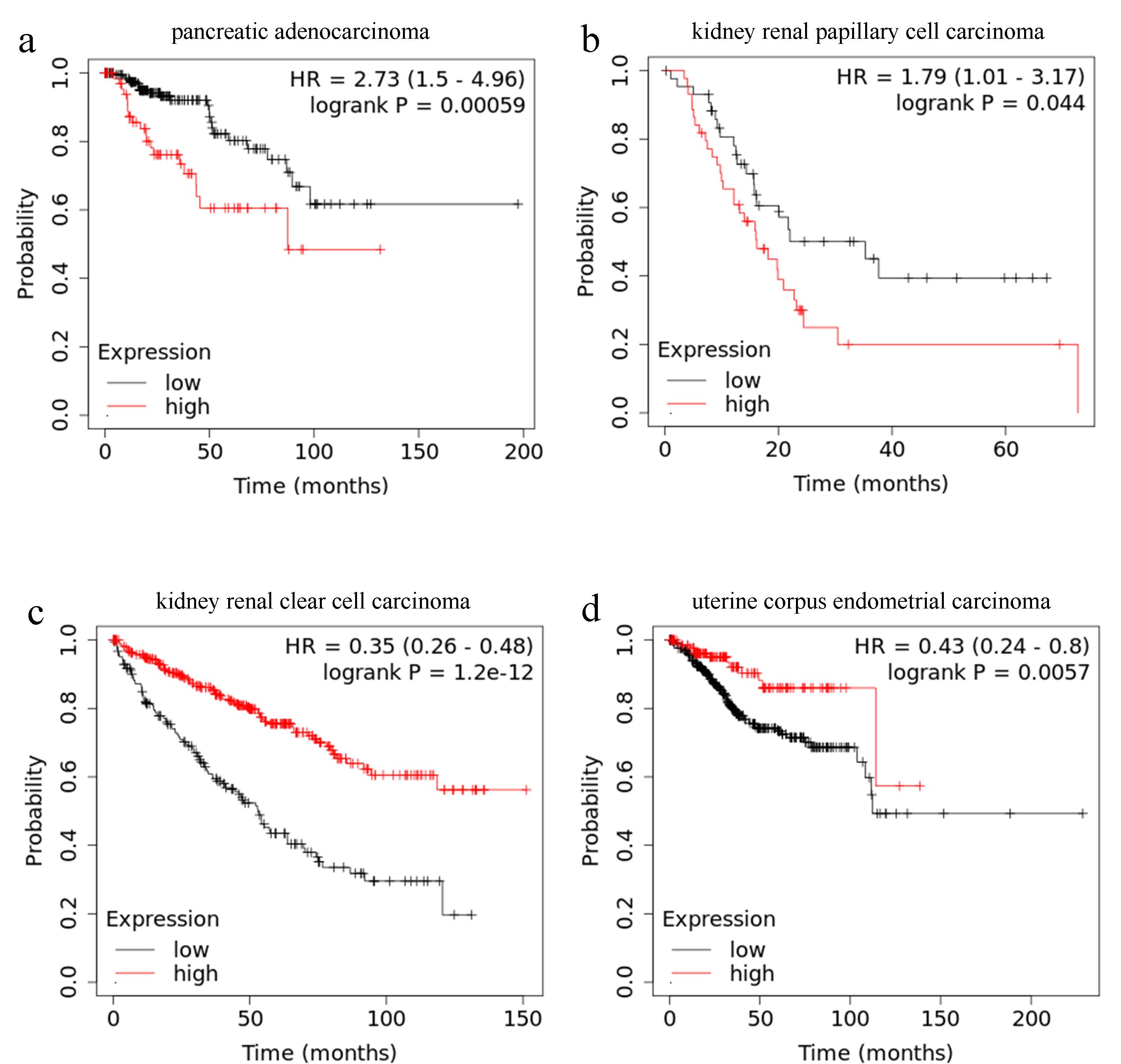

Supplement: Supplementary file 2 — Additional file 2 SF.1 Kaplan-Meier survival curves for cancer patients from KM Plotter. (a) Pancreatic adenocarcinoma; (b) kidney renal papillary cell carcinoma; (c) kidney renal clear cell carcinoma; (d) uterine corpus endometrial carcinoma. [file 12885_2020_7108_MOESM2_ESM.tif]
